# Loss of phosphodiesterase 4D mediates acquired triapine resistance via Epac-Rap1-Integrin signaling

**DOI:** 10.18632/oncotarget.11821

**Published:** 2016-09-02

**Authors:** Walter Miklos, Petra Heffeter, Christine Pirker, Sonja Hager, Christian R. Kowol, Sushilla van Schoonhoven, Mirjana Stojanovic, Bernhard K. Keppler, Walter Berger

**Affiliations:** ^1^ Department of Medicine I, Institute of Cancer Research and Comprehensive Cancer Center, Medical University of Vienna, A-1090 Vienna, Austria; ^2^ Institute of Inorganic Chemistry, University of Vienna, A-1090 Vienna, Austria; ^3^ Research Platform “Translational Cancer Therapy Research”, University Vienna and Medical University Vienna, Vienna, Austria

**Keywords:** triapine-resistance, phosphodiesterase, Epac, Rap1, integrin

## Abstract

Triapine, an anticancer thiosemicarbazone, is currently under clinical investigation. Whereas promising results were obtained in hematological diseases, trials in solid tumors widely failed. To understand mechanisms causing triapine insensitivity, we have analysed genomic alterations in a triapine-resistant SW480 subline (SW480/tria). Only one distinct genomic loss was observed specifically in SW480/tria cells affecting the phosphodiesterase 4D (*PDE4D*) gene locus. Accordingly, pharmacological inhibition of PDE4D resulted in significant triapine resistance in SW480 cells. Hence, we concluded that enhanced cyclic AMP levels might confer protection against triapine. Indeed, hyperactivation of both major downstream pathways, namely the protein kinase A (PKA)-cAMP response element-binding protein (Creb) and the exchange protein activated by cAMP (Epac)-Ras-related protein 1 (Rap1) signaling axes, was observed in SW480/tria cells. Unexpectedly, inhibition of PKA did not re-sensitize SW480/tria cells against triapine. In contrast, Epac activation resulted in distinct triapine resistance in SW480 cells. Conversely, knock-down of Epac expression and pharmacological inhibition of Rap1 re-sensitized SW480/tria cells against triapine. Rap1 is a well-known regulator of integrins. Accordingly, SW480/tria cells displayed enhanced plasma membrane expression of several integrin subunits, enhanced adhesion especially to RGD-containing matrix components, and bolstered activation/expression of the integrin downstream effectors Src and RhoA/Rac. Accordingly, integrin and Src inhibition resulted in potent triapine re-sensitization especially of SW480/tria cells. In summary, we describe for the first time integrin activation based on cAMP-Epac-Rap1 signaling as acquired drug resistance mechanism. combinations of triapine with inhibitors of several steps in this resistance cascade might be feasible strategies to overcome triapine insensitivity of solid tumors.

## INTRODUCTION

Conversion of ribonucleotides to deoxyribonucleotides is the rate-limiting step during DNA synthesis catalyzed by ribonucleotide reductase (RR) [1]. Because of the high proliferation rate of tumor cells, RR is overexpressed in many different cancer types and represents a potent target in anticancer treatment [2]. RR is composed of the catalytic R1 and the enzymatic R2 subunit. The latter comprises a tyrosyl radical within an iron-containing center essential for the enzymatic activity [3]. Consequently, iron depletion in cancer cells leads to deoxyribonucleotide deprivation and further to cell cycle arrest and apoptosis. Therefore, iron-chelating drugs have been developed as targeted anticancer drugs. Several prominent representatives belong to the class of thiosemicarbazones (TSC) [1], with 3-aminopyridine-2-carboxaldehyde thiosemicarbazone (triapine or 3-AP) as the best studied example. While promising results for triapine were obtained in clinical studies concerning hematological diseases [2, 4], the one in solid tumors widely failed [5-8]. However, the mechanisms underlying triapine insensitivity of solid tumors are widely unknown.

Intrinsic and acquired anticancer drug resistance might be based on overexpression of ATP-binding cassette (ABC) drug efflux pumps causing a phenomenon termed multi-drug resistance (MDR). With regard to ABC-transporters, others and we have shown that triapine-selection might cause ABCB1 (also known as P-glycoprotein) overexpression [9, 10]. However, triapine accumulation was not reduced significantly in ABCB1-overexpressing cells and ABCB1 inhibition failed to significantly re-sensitize towards triapine. This argues against ABCB1 as a major triapine-resistance factor [10]. Alternatively, drug resistance might be based on hyperactivation of different survival and anti-apoptosis pathways. One of the involved molecules, attracting increasing attention during the last years, represents cyclic adenosine monophosphate (cyclic AMP, cAMP) [11]. cAMP is one of the most abundant second messengers regulating various physiological processes like cell survival, differentiation, proliferation and apoptosis [12]. cAMP is catalyzed from ATP via adenylate cyclase activated by G protein-coupled receptors [13]. The negative regulators of cAMP belong to the family of phosphodiesterases (PDE), especially PDE4, which hydrolyze cAMP to AMP [14, 15]. Enhanced cAMP signaling has recently been identified as a major player in resistance to cytotoxic drugs, but also for example in vemurafenib therapy failure of BRAF-mutant melanoma [11].

One major downstream target of cAMP is cAMP-dependent protein kinase A (protein kinase A, PKA) [16], a serine/threonine kinase consisting of two catalytic and two regulatory subunits [17]. Binding of the second messenger to the regulatory subunits leads to dissociation of the complex and the free catalytic subunits phosphorylate a variety of proteins [18]. One PKA substrate is the transcription factor cAMP-responsive element-binding protein (Creb). Subsequent, Creb binds as homo- or heterodimers to cAMP-responsive elements within target gene promoters [19], thus activating transcription of several growth and survival genes including microphthalmia-associated transcription factor (MITF) [20], bcl-2 [21], and cyclin D1 [22].

Besides PKA, another downstream target of cAMP, namely exchange protein activated by cAMP (Epac), was more recently discovered [23]. Epac acts as cAMP-activated Rap guanine-nucleotide-exchange factor for the GTPase Ras-related protein 1 (Rap1), which it activates independently of PKA [24]. Rap1 signaling leads to various cellular responses including integrin-mediated cell adhesion [25], as well as activation of the mitogen-activated protein kinase signaling pathway [26], Src [27], and downstream GTPases of the Rho/Rac family via a crosstalk between Src and focal adhesion kinase (FAK) [28]. Accordingly, Almahariq et al. have shown that Epac plays an important role in pancreatic cancer cell migration and invasion via altered integrin expression [29].

In the present study, we have uncovered a key role of the cAMP signaling pathway in acquired triapine resistance. An Epac-Rap1-integrin survival program was identified as the responsible downstream mediator. These findings offer the chance for synergistic combination treatment schemes involving triapine.

## RESULTS

### Genomic characterization of triapine-resistant SW480 cells

**Figure 1:**
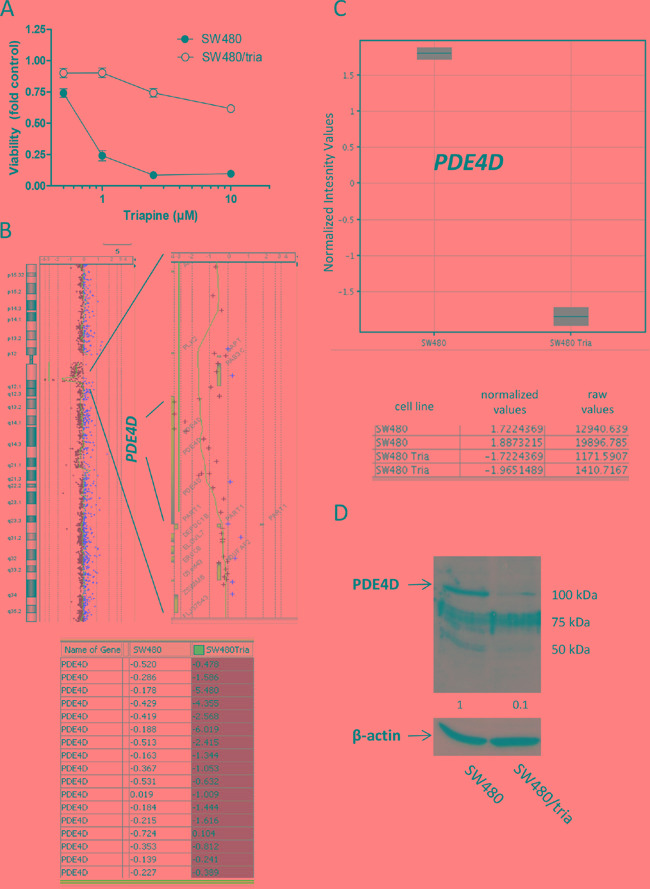
Characterization of a colon cancer cell line with acquired triapine resistance **A.** SW480 and SW480/tria cells were treated with the indicated concentrations of triapine. After 72 h treatment, cell viability was determined by MTT assay. The values given are means and standard deviations (SD) of one representative experiment out of three performed in triplicate. **B.** Gene dose changes of SW480/tria in comparison to SW480 cells were investigated by indirect aCGH (compare Supplementary Figure S1) using Cy5- and Cy3-labeled DNA, respectively. Results for whole chromosome 5 (left) are opposed to an enlarged region at 5q12 (right) containing the *PDE4D* gene. Each (+) indicates the position of one oligonucleotide within this region. Log2 ratios of Cy5-labeled SW480/tria and Cy3-labeled SW480 DNA of all *PDE4D* oligonucleotides on the microarray are listed at the bottom. **C.** mRNA expression levels for *PDE4D* in SW480 and SW480/tria cells were assessed by whole genome gene expression microarrays. Three independent *PDE4D* oligonucleotides were spotted on the array and gave comparable results. Data for oligonucleotide A_33_P3389653, recognizing all *PDE4D* mRNA splice variants, are shown. Normalized values for two replicates of SW480 and SW480/tria cells indicate massive downregulation of *PDE4D* mRNA in the SW480/tria as compared to the parental cell line. Raw values depict the absolute dye intensity (Cy3 or Cy5) measured for the *PDE4D* oligonucleotide on the microarray. **D.** PDE4D expression using total protein extracts of SW480 vs. SW480/tria cells was evaluated by Western blotting. β-actin was used as loading control.

A triapine-resistant SW480 subline (SW480/tria) was generated as described by Miklos et al [10]. Cytotoxicity assays were performed to prove insensitivity against triapine (Figure 1A) and a >56-fold resistance (at IC_50_) in comparison to the parental cell line was detected. To identify molecular factors contributing to triapine resistance, genome wide gene dose changes (gains or losses) were analyzed by direct and indirect aCGH. While multiple chromosomal changes were obvious in the parental cell line (Supplementary Figure S1A), indirect aCGH of SW480/tria versus SW480 cells indicated that triapine selection induced only minor further DNA dose changes (Supplementary Figure S1B). Distinct genomic gains in SW480/tria as compared to the parental cells were completely missing. In contrast, the resistant subline harbored a specific loss at chromosome 5q12 (Figure 1B). The deleted region affected only one gene, namely phosphodiesterase 4D (*PDE4D*). As expected, gene loss resulted in massive downregulation of the respective *PDE4D* mRNA (Figure 1C) and protein expression (Figure 1D).

### PDE4D inhibition protects against triapine in parental SW480 cells

**Figure 2:**
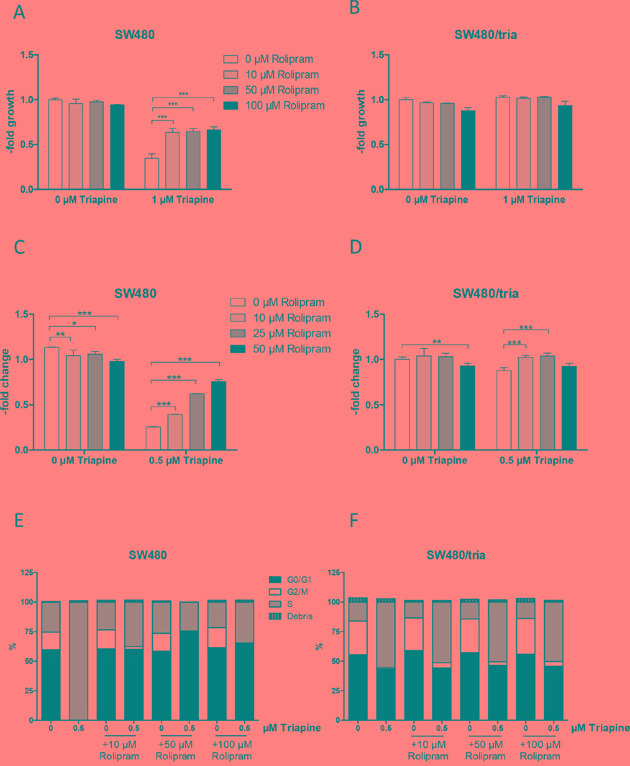
Impact of PDE4D inhibition on triapine response in SW480 and SW480/tria cells **A** and **B.** Cell viability of SW480 (A) and SW480/tria (B) cells treated for 72 h with the PDE4D inhibitor rolipram at the indicated concentrations alone and in combination with 1 μM triapine was determined by MTT assay. The values given are means and SD of one representative experiment out of three performed in triplicate. Statistical analysis was performed by two-way ANOVA (***P < 0.001). **C** and **D.** Colony formation potency of SW480 (C) and SW480/tria cells (D) treated with the indicated concentrations of rolipram alone and in combination with 0.5 μM triapine was evaluated. After 10 days of drug exposure cells were stained with crystal violet and quantified microscopically. The values given are means and SD of two experiments in duplicate. Statistical analysis was performed by Student's t-test (*P < 0.05, **P < 0.01, ***P < 0.001). **E** and **F.** Cell cycle distribution of SW480 (E) and SW480/tria cells (F) was determined after 24 h exposure to the indicated concentrations of triapine alone and in combination with rolipram. Propidium iodide staining and flow cytometric measurements were performed and percentages of cells in G0/G1, S, and G2/M phases are indicated.

Consequently, we hypothesized that PDE4D augments triapine-mediated anticancer activity probably via down-regulation of cAMP. To support this assumption, we investigated whether the small molecule PDE4 inhibitor rolipram protects SW480 cells against triapine. Indeed, cell viability assays (72 h exposure) proved that PDE4 inhibition by rolipram resulted in decreased sensitivity of SW480 cells (Figure 2A) as well as HCT-116 cells (Supplementary Figure S2A) to triapine. In contrast, no significant effects were seen in the already triapine-resistant SW480/tria cells (Figure 2B). Long-term colony formation assay (10 days exposure) confirmed this protective effect in parental SW480 cells already at a lower triapine concentration (0.5 μM; Figures 2C, 2D and Supplementary Figures S2B, S2C). As triapine is a known ribonucleotide reductase inhibitor [30], we further analysed the cell cycle distribution in the drug combination setting. Interestingly, the almost complete S-phase arrest induced by 0.5 μM triapine in SW480 cells was distinctly abolished by rolipram (Figure 2E). While reduction of the G2/M subpopulation by triapine was also detected in SW480/tria cells, the massive S-phase arrest was missing. Furthermore, co-treatment with rolipram only marginally reversed the G2/M-phase loss induced by triapine (Figure 2F).

### The cAMP-PKA-Creb signal axis is not a major regulator of PDE4D-promoted triapine response

**Figure 3:**
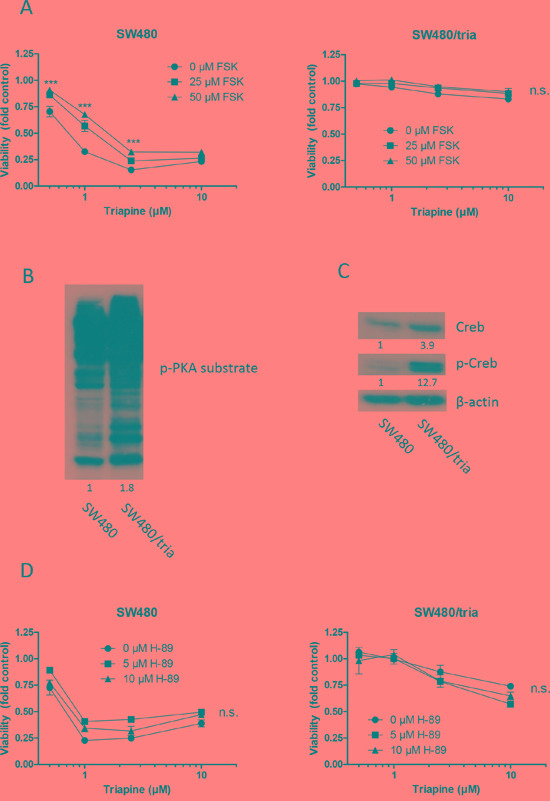
The PKA-Creb signaling axis is not involved in triapine resistance **A.** SW480 and SW480/tria cells were treated with triapine alone and in combination with forskolin (FSK, adenylate cyclase activator) at the indicated concentrations. After 72 h treatment, cell viability was determined by MTT assay. The values given are means and SD of one representative experiment out of three performed in triplicate. Statistical analysis was performed by two-way ANOVA (***P < 0.001). **B** and **D.** Phosphorylation levels of multiple PKA substrates (B) and Creb (C) in total protein extracts of SW480 vs. SW480/tria cells was evaluated by Western blotting. β-actin was used as loading control. **D.** SW480 and SW480/tria cells were treated with triapine alone and in combination with H-89 (PKA inhibitor). After 72 h treatment, cell viability was determined by MTT assay. The values given are means and SD of one representative experiment out of three performed in triplicate.

One of the major cellular signaling pathways activated by cAMP is the PKA-Creb module [16]. Therefore, we investigated whether alterations in the cAMP-PKA-Creb pathway were responsible for triapine resistance mediated by PDE4D loss. Indeed, stimulation of cAMP with forskolin significantly attenuated triapine response in SW480 cells but not in the triapine-selected subline (Figure 3A). Forskolin as single drug did not markedly alter viability of SW480 cells but slightly reduced the one of SW480/tria cells (Supplementary Figure S3A). Furthermore, hyperactivation of PKA in SW480/tria cells compared to the parental cell line was demonstrated by a strong hyperphosphorylation of PKA substrates (Figure 3B). More specifically, expression of the major PKA downstream transcription factor Creb was slightly enhanced and its activating phosphorylation at serine 133 massively increased in the triapine-resistant subline (Figure 3C). Thus, we hypothesized that inhibition of the PKA/Creb signal by PKA inhibitor H-89 should re-sensitize SW480/tria cells against triapine. The inhibitor alone had no major influence on cell viability in both cell lines (Supplementary Figure S3B). Surprisingly, however, co-application with H-89 did not significantly sensitize SW480/tria cells against triapine and even tended to protect the parental cell line (Figure 3D) despite clear-cut reduction of Creb phosphorylation in both cell models (Supplementary Figure S3C). This demonstrates that the PKA-Creb signal axis is not the major player involved in cAMP-mediated triapine resistance.

### The cAMP-Epac-Rap1 signal axis distinctly contributes to acquired triapine resistance

**Figure 4:**
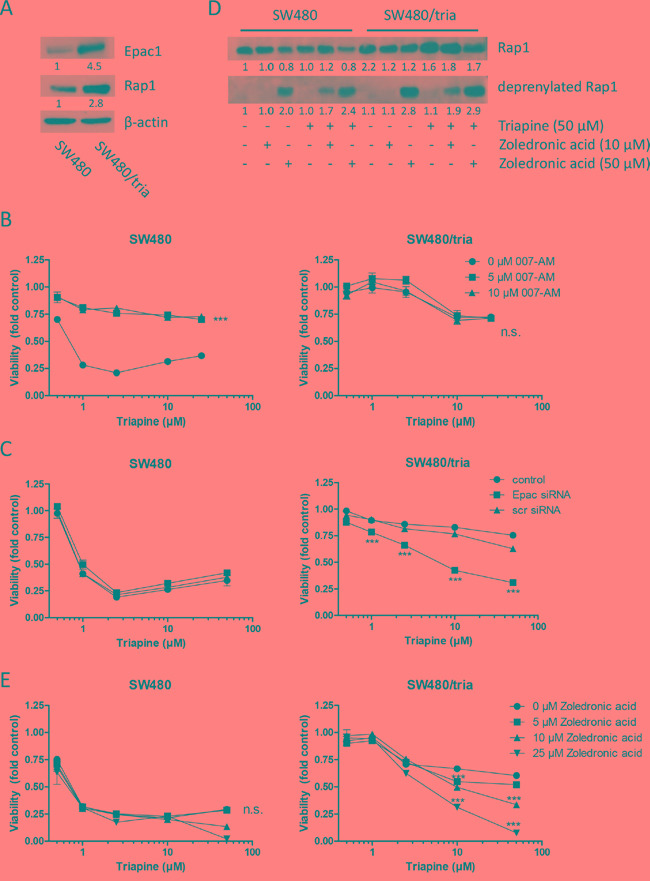
The Epac-Rap1 signaling axis is involved in triapine resistance **A.** Expression of total Epac1 and Rap1 in protein extracts of SW480 vs. SW480/tria cells. **B.** SW480 and SW480/tria cells were treated with the indicated concentrations of triapine alone and in combination with 007-AM (Epac activator). After 72 h treatment cell viability was determined by MTT assay. The values given are means and SD of one representative experiment out of three performed in triplicate. Statistical analysis was performed by two-way ANOVA (***P < 0.001). **C.** SW480 and SW480/tria cells were left untreated (control) or exposed to either *Epac* or scrambled (scr) siRNA (both 25 μM) for 48 h and treated with the indicated concentrations of triapine. After 72 h treatment, cell viability was determined by MTT assay. The values given are means and SD of one representative experiment out of three performed in triplicate. Statistical analysis was performed by two-way ANOVA (***P < 0.001). **D.** Levels of total and deprenylated Rap1 in SW480 as compared to SW480/tria cells treated with the indicated drugs for 24 h were determined by Western blotting. β-actin was used as loading control. **E.** SW480 and SW480/tria cells were treated with the indicated concentrations of triapine alone and in combination with zoledronic acid (inhibiting Rap1 prenylation). After 72 h treatment, cell viability was determined by MTT assay. The values given are means ± SD of one representative experiment out of three performed in triplicate. Statistical analysis was performed by two-way ANOVA (***P < 0.001).

An alternative target of cAMP is Epac [23], a guanine nucleotide exchange factor selectively activating the Rap1 protein [24]. Therefore, we investigated whether Epac and Rap1 are involved in acquired triapine resistance. Indeed, both Epac and Rap1 were markedly overexpressed in the triapine-resistant SW480 subline (Figure 4A). Activation of Epac by the cell-permeable activator 007-AM led to a massively reduced triapine response selectively in SW480 but not in SW480/tria cells (Figure 4B). Accordingly, Epac knock-down by siRNA (Supplementary Figure S4A) led to significant re-sensitization of the resistant subline to triapine, whereas no effect was seen in the parental SW480 cells (Figure 4C). In addition to overexpression, Rap1 was clearly hyper-activated in the triapine-resistant subline (Supplementary Figure S4B). Furthermore, triapine treatment led to a further increase of Rap1 expression levels in SW480/tria but not in parental SW480 cells (Figure 4D). Rap1 needs to be prenylated for correct localization and activation [31]. Accordingly, deprenylation of Rap1 as a consequence of mevalonate pathway inhibition by zoledronic acid led to higher amounts of deprenylated Rap1 in SW480/tria cells especially when co-administered with triapine (Figure 4D). SW480/tria cells were slightly but significantly hypersensitive against zoledronic acid as a single drug in comparison to the parental cell line (Supplementary Figure S4C). Furthermore, Rap1 inhibition by zoledronic acid resulted in re-sensitization of SW480/tria cells to triapine but had almost no impact in parental cells (Figure 4E). This synergistic effect was confirmed by combination index values <0.8 especially in the resistant cell model (Supplementary Figure S4D). These data strongly indicate that triapine resistance is mediated at least in part via the cAMP-Epac-Rap1 signal axis.

### Epac-Rap1-mediated triapine resistance involves integrin alterations

**Figure 5:**
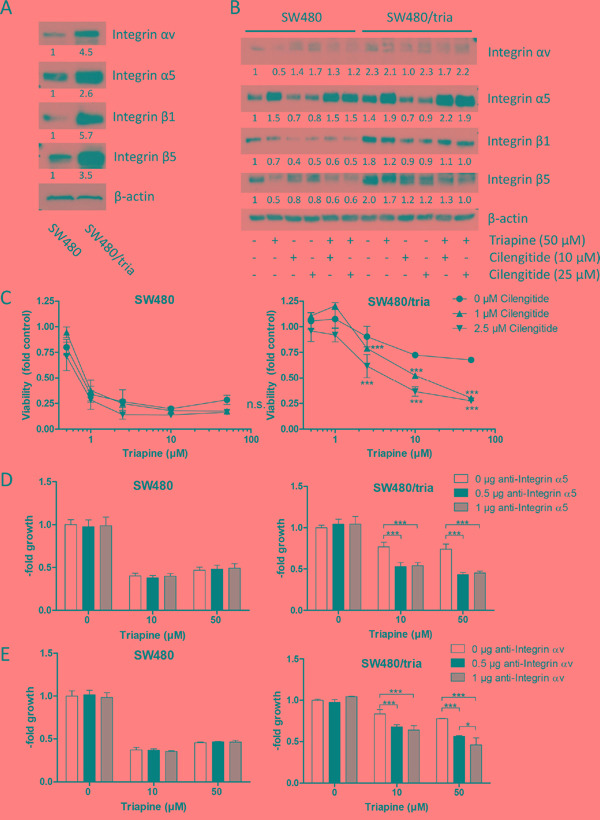
Role of integrins in acquired triapine resistance of SW480/tria cells **A.** and **B.** Levels of integrin *αv*, α5, β1, and β5 in plasma membrane-enriched protein fractions (A) and in total protein extracts prepared from SW480 and SW480/tria cells treated as indicated (B) were determined by Western blotting. β-actin was used as loading control. **C.** SW480 and SW480/tria cells were treated with the indicated concentrations of triapine alone and in combination with cilengitide. After 72 h treatment, cell viability was determined by MTT assay. The values given are means and SD of one representative experiment out of three performed in triplicate. Statistical analysis was performed by two-way ANOVA (***P < 0.001). **D** and **E.** SW480 and SW480/tria cells were treated with the indicated concentrations of triapine alone and in combination with specific inhibitory antibodies for integrin α5 (D) or integrin *αv* (E). After 72 h treatment, cell viability was determined by MTT assay. The values given are means and SD of three independent experiments performed in triplicates. Statistical analysis was performed by two-way ANOVA (*P < 0.05, ***P < 0.001).

Next, we aimed to clarify how the Epac-Rap1 signaling cascade mediates triapine resistance. Therefore, we investigated the role of integrins, major downstream targets of Rap1 [32], in acquired triapine resistance. Expression of several integrin α and β subunits was analyzed by Western blot in plasma membrane-enriched fractions of SW480 as compared to SW480/tria cells. The amounts of membrane-associated integrin subunits *αv*, α5, β1 and β5 were distinctly enhanced in SW480/tria as compared to SW480 cells (Figure 5A). Integrin β1 overexpression was also confirmed by immunhistochemical staining in the SW480/tria as compared to the SW480 xenograft *in vivo* (Supplementary Figure S5A, S5B). Consequently, we analyzed the impact of triapine treatment alone and in combination with cilengitide, a cyclic RGD-mimetic peptide and integrin *αv* inhibitor [33], on integrin expression in our resistance model using total protein lysates. Generally, upregulation of integrin expression in the resistant subline was somewhat lower but still distinct in the total protein lysates as compared to the cell membrane fractions, indicating more efficient cell membrane targeting of integrins in the triapine-resistant subline (Figure 5B versus 5A, respectively). Interestingly, triapine exposure for 24 h resulted in a selective and distinct up-regulation of integrin α5 expression in both cell lines (Figure 5B). In contrast, it had no further stimulatory effects on the expression of integrin *αv* and the β-subunits. To investigate the mechanisms underlying massive expression stimulation of integrin α5 in response to triapine, real-time PCR analyses for the respective *ITGA5* mRNA were performed (Supplementary Figure S5C). While upregulation of *ITGA5* mRNA was only about two-fold in SW480/tria as compared to the parental SW480 cells, short-term triapine exposure induced massive *ITGA5* mRNA upregulation in both cell models. This suggests that transcriptional and post-transcriptional mechanisms are involved in the regulation of integrin expression/activity by triapine.

Integrin *αv* inhibition by cilengitide led to significant triapine re-sensitization of SW480/tria cells, while no synergism was detected in the parental cell line (Figure 5C; Supplementary Figure S5E). Additionally, SW480/tria cells were per se hypersensitive towards cilengitide when used as single drug (Supplementary Figure S5D). To dissect the role of integrin α5 and *αv* in acquired triapine resistance, combination experiments of triapine with specific blocking antibodies for these integrin subunits were conducted (Figure 5D and 5E). Interestingly, both antibodies significantly reversed acquired triapine resistance in SW480/tria cells but were widely inactive in the parental cell model. Hence, we conclude that both integrin α subunits play a pivotal role in acquired triapine resistance.

### Integrin-mediated cell adhesion is altered by triapine selection

**Figure 6:**
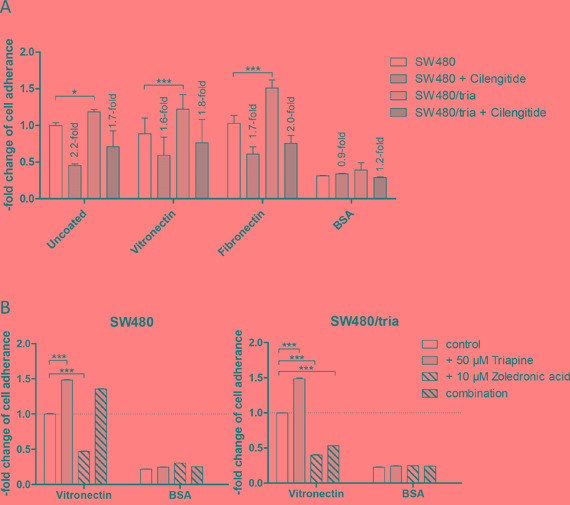
Integrin-mediated cell adhesion in SW480 as compared to SW480/tria cells **A** and **B.** Adhesion of SW480 and SW480/tria cells to the indicated substrates and the impact of 1 μM cilengitide (A) and 50 μM triapine alone and in combination with 10 μM zoledronic acid (B) was analyzed as described in the Material and Methods section. All experiments were repeated three times in duplicates. The means and SD of all three experiments (A) and one representative experiment out of three delivering widely comparable results (B) are shown. Statistical analysis was performed by two-way ANOVA (*P < 0.05, ***P < 0.001).

Integrins are heterodimeric cell surface receptors for ligands in the extracellular matrix leading to activation of intracellular signaling cascades to promote adhesion, migration, proliferation, and survival [34]. To further elucidate altered integrin signaling in triapine resistance, cell adhesion assays with SW480 and SW480/tria cells were performed (Figure 6A). In accordance with the increased expression of several integrin subunits, the triapine-resistant subline was able to adhere moderately but significantly more efficiently to uncoated cell culture plastic as compared to the parental cell line. On surfaces coated with ligands for RGD-binding integrins, namely vitronectin and fibronectin, this difference was distinctly enhanced. In contrast, adhesion to the BSA-coated surface was unaltered in SW480/tria as compared to SW480 cells. Blockade of integrins by cilengitide treatment resulted in reduced cell adhesion efficacy to culture plastic as well as vitronectin and fibronectin, while there was no significant effect in case of BSA. These effects were stronger in the resistant subline in case of the RGD-containing ligands. Furthermore, short-term triapine treatment of both SW480 as well as SW480/tria cells resulted in enhanced adhesion competence to vitronectin but not BSA corresponding to the changes in integrin α5 expression observed in Western blot analyses (Figure 6B; compare Figure 5B). Adherence of both cell models to vitronectin was massively inhibited by Rap1 blockade via zoledronic acid. Presence of triapine abrogated this effect almost completely in the parental but only marginally in the triapine-resistant subline (Figure 6B). This indicates dominance of the mevalonate pathway/Rap1 signaling module in triapine-mediated regulation of integrin dynamics in SW480/tria but not SW480 cells.

### Possible downstream effectors of Epac-Rap1-integrin-mediated triapine resistance

**Figure 7:**
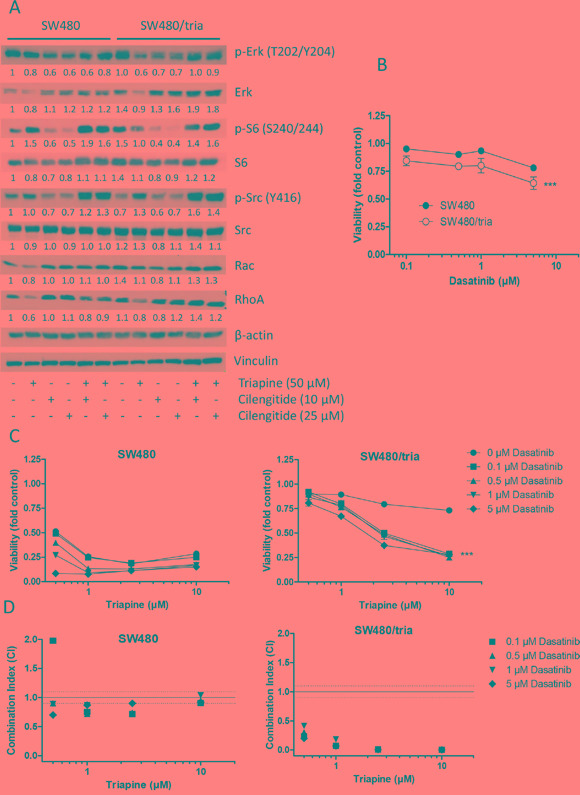
Activation of integrin-mediated downstream signaling pathways in SW480 as compared to SW480/tria cells **A.** Activating phosphorylation of Erk (T202/Y204), S6 (S240/244) and Src (Y416) as well as expression of the integrin downstream GTPases Rac and RhoA in SW480 and SW480/tria cells treated with either triapine and cilengitide alone or in combination at the indicated concentrations were determined by Western blot. β-actin and vinculin were used as loading controls. **B** and **C.** SW480 and SW480/tria cells were treated with the indicated concentrations of dasatinib (Src inhibitor) alone (B) and in combination with triapine (C). After 72 h treatment, cell viability was determined by MTT assay. The values given are means and SD of one representative experiment out of three performed in triplicate. Statistical analysis was performed by two-way ANOVA (***P < 0.001). **D.** Combination indices (CI) for the 72 h analyses shown in C were calculated using CalcuSyn software. CI < 0.9, CI = 0.9 - 1.1 or CI > 1.1 represent synergism, additive effects and antagonism, respectively.

Ligand binding by integrin mediates activation of multiple intracellular signaling pathways including MEK/ERK, PI3K/AKT, and Src, as well as the downstream GTPases RhoA and Rac [34]. To elucidate possible mechanisms underlying Epac/Rap1/integrin-mediated triapine resistance, regulation of these pathways by triapine without and with cilengitide was investigated in SW480 as compared to SW480/tria cells. Phosphorylation of ERK, S6, and Src was used as indicator for activation of the respective pathways (Figure 7A). While both drugs and their combinations had massive impacts on the phosphorylation of ERK, the alterations were widely comparable between SW480 and SW480/tria cells. Phosphorylation of S6 was enhanced in SW480/tria cells as compared to the parental cell line. Triapine as single agent induced S6 phosphorylation in SW480 but inhibited hyperphosphorylation in SW480/tria cells. In both cell models cilengitide alone massively blocked S6 phosphorylation whereas combination with triapine restored PI3K/AKT pathway activity. The most interesting differences were found in case of Src. Basal phosphorylation was marginally reduced in SW480/tria, compared to SW480 cells (Figure 7A). Triapine significantly stimulated the level of p-Src in SW480/tria cells while no effect was seen in the parental cells. Cilengitide as single drug reduced Src phosphorylation in SW480 and SW480/tria cells while combination of both drugs resulted in enhanced Src phosphorylation especially in the resistant subline. Moreover, the triapine-resistant subline was moderately but significantly hypersensitive against the Src-inhibitor dasatinib (Figure 7B). Furthermore, Src inhibition led to significant triapine re-sensitization of SW480/tria cells (Figure 7C). The synergistic effect of triapine and dasatinib co-exposure was also confirmed by the respective combination indices (Figure 7D). At low μM triapine concentrations, however, dasatinib also re-sensitized the parental SW480 cells against triapine (Supplementary Figure S6). This indicates that Src phosphorylation is one player underlying acquired as well as intrinsic triapine resistance. Additionally, expression of two additional downstream targets of integrins, namely the GTPases Rac and RhoA, was investigated. Especially basal Rac expression was clearly enhanced in triapine-resistant cells compared to the parental cell line. While triapine treatment as single agent reduced Rac and RhoA expression in both cell lines, combination with cilengitide led to enhanced Rac and RhoA levels selectively in SW480/tria cells (Figure 7A). These data suggest cooperation of several integrin-mediated signaling pathways in acquired triapine resistance.

## DISCUSSION

Triapine, the most prominent anticancer thiosemicarbazone, showed promising results in clinical trials against hematological diseases [2, 4] but failed in solid tumors due to unknown reasons so far [5-8]. To investigate in depth the mechanism of acquired triapine resistance, we utilized the human colon carcinoma cell line SW480 and its triapine-resistant subline SW480/tria [10]. Previously, we have reported on ABCB1 overexpression in this triapine-resistant SW480 subline and the corresponding cross-resistance of SW480/tria cells against several classical chemotherapeutics and substrates of ABCB1-mediated drug efflux. However, inhibition of ABCB1 did not result in significant re-sensitization to triapine suggesting that ABCB1 overexpression is not the main resistance mechanism in this cell model but rather a general consequence of stress response [10]. Therefore, we aimed to identify additional triapine resistance mechanisms by screening for genome-wide gene dose alterations using aCGH. Interestingly, only minimal additional gene dose changes were caused by long-term triapine selection with the exception of a distinct loss at the chromosomal region 5q12 in SW480/tria cells. This homozygous deletion selectively affected the *PDE4D* gene.

In the literature, alterations in phosphodiesterase 4 family members and the resulting changes in cAMP levels were connected with oncogenic but also tumor-suppressive functions. With regard to pro-tumorigenic activities, PDE4 inhibitors were reported to inhibit proliferation, migration, and progression of several solid tumors as well as hematological malignancies probably via cAMP-PKA signaling [35-38]. Accordingly, the cancer-preventive and therapeutic effects of several natural products including curcumin and resveratrol were attributed to PDE4D inhibition [39, 40]. Furthermore, there is strong evidence for PDE4 to promote angiogenesis via hypoxia-inducible factor in lung cancer [41]. In contrast to these indications of pro-tumorigenic functions, repeatedly homozygous deletions of the *PDE4D* gene in several malignancies have been reported [42, 43]. For example, Nancarrow et al. described that homozygous deletion of *PDE4D* resulted in a tumor suppressor phenotype in esophageal adenocarcinoma [44]. Microdeletions at the *PDE4D* gene locus in diverse solid tumors resulted in enhanced gene expression and sensitivity towards *PDE4D* knock-down [43]. Moreover, McEwan et al. reported that combination of the PDE4 inhibitor rolipram and low doses of the adenylate cyclase activator forskolin resulted in colon cancer cell growth inhibition implicating that these cells are addicted to maintenance of low cAMP concentrations in a compartment that is regulated by PDE4 [45]. In the present study, deletion of major parts of the *PDE4D*-coding region as a consequence of triapine selection resulted in almost complete blockade of gene expression at the mRNA and protein level. Interestingly, this was not accompanied by altered tumorigenicity and growth dynamics as xenograft in SCID mice arguing against a major oncogenic or tumor-suppressive role of PDE4D in the SW480 colon cancer cell model. In contrast, however, we were able to proof an important contribution of PDE4D loss to the triapine resistance phenotype of SW480/tria cells.

PDE4D loss in our resistant cell model resulted in upregulation of cAMP and its major downstream signaling cascade, namely the PKA-Creb axis [16]. Accordingly, PDE4D inhibition in the parental cell line via rolipram and stimulation of cAMP with forskolin resulted in triapine resistance in the parental cell line but did not affect SW480/tria cells. Hence, we hypothesized that this signal module might be the driver for acquired triapine resistance. In line with that, several indications for a role of PKA-Creb activation in oncological therapy failure have been reported. For instance, tamoxifen resistance in hormone receptor-positive and trastuzumab resistance in HER2-positive breast cancer might be mediated via the PKA-Creb axis [46-48]. Moreover, doxorubicin resistance has been associated with hyperactivation of Creb in hepatocellular carcinoma [49] and mesothelioma [50]. In accordance, our triapine-resistant SW480 cells displayed distinct cross-resistance against doxorubicin [10]. However, on the one hand, it has to be kept in mind that SW480/tria cells are overexpressing ABCB1 readily excreting doxorubicin. On the other hand, it is well described that PKA-Creb signaling results in increased ABCB1 expression and inhibition of this pathway might reverse ABCB1-mediated multi-drug resistance [51, 52]. Furthermore, we have demonstrated previously that the markedly enhanced expression of protein kinase C (PKC) in SW480/tria cells contributes to ABCB1 overexpression [10]. Interestingly, strong evidence exists for a crosstalk between PKC and PKA-Creb signaling pathways [53] suggesting that PDE4D loss might play a role in ABCB1 overexpression in SW480/tria cells. Surprisingly, however, inhibition of PKA with H-89 did not significantly re-sensitize SW480/tria cells against triapine. Hence, we had to conclude that the distinctly enhanced PKA-Creb signaling axis in SW480/tria cells is not a major player in acquired triapine resistance.

Due to this fact, we extended our investigation to the more recently discovered second arm of cAMP signaling, namely the Epac-Rap1 axis, which is also hyper-activated in SW480/tria cells. Indeed, Epac activation by 007-AM resulted in triapine resistance of the parental cell line, whereas *Epac* knock-down and blockade of Rap1 by the prenylation inhibitor zoledronic acid [54, 55] re-sensitized SW480/tria against triapine. In contrast to the PKA-Creb axis, the Epac-Rap1 signaling module was unequivocally linked to pro-tumorigenic and resistance-mediating mechanisms. Thus, enhanced Epac expression plays an important role in migration and metastasis in melanoma [56] and pancreatic cancer [29]. Moreover, Onodera et al. described that the Epac-Rap1 signaling pathway regulates glucose uptake and metabolism and therefore promotes oncogenesis [57]. Accordingly, knock-down of *Rap1A* increased sensitivity to taxol in non-small cell lung cancer [58]. It has to be mentioned that Epac represents only one out of several Rap1 GEFs, including for example C3K (Crk SH3-domain-binding guanine nucleotide-releasing factor, also known as RAPGEF1), calcium and diacylglycerol (CalDAG)-GEFs and PDZ-GEF1/2 [59]. Hence, considering the tissue-specific expression of Epac [60], one might hypothesize that also other mechanisms for Rap1 activation in triapine resistance might exist.

Interestingly, the adhesion-mediating but also tumor-promoting functions of cAMP-Rap1 signaling have been repeatedly linked to its impact on integrin expression and activity [61, 62]. Therefore, we decided to investigate whether triapine resistance is based on integrin-mediated survival signaling. Indeed, we observed an increased basal expression of several oncogenic RGD-binding integrin subunits including *αv*, α5, β1, and β5 in the SW480/tria cells especially in plasma membrane-enriched protein fractions. Additionally, we detected a selective stimulation of integrin α5 expression on the mRNA and protein level by short-term triapine treatment. Interestingly, the respective *ITGA5* mRNA was only about two-fold enhanced in SW480/tria as compared to SW480 cells. However, short-term triapine treatment enhanced *ITGA5* mRNA around ten-fold in both cell models while integrin *αv* was widely unresponsive at mRNA (data not shown) and protein level. This implicates cooperation between broad integrin activation by upstream mechanisms involving Epac-Rap1 signaling in acquired triapine resistance and, additionally, transcriptional upregulation of integrin α5-containing integrin dimers due to an immediate triapine response. The underlying mechanisms are currently dissected in ongoing experiments.

In agreement with enhanced integrin expression, cell adherence of the resistant subline was moderately more efficient to cell culture plastic but distinctly enhanced when using ligands for RGD-binding integrins, namely vitronectin and fibronectin. Furthermore, this adhesion was reduced by the integrin inhibitor cilengitide and the Rap1-deprenylating agent zoledronic acid at higher potency in case of the resistant subline. Accordingly, integrin inhibition by cilengitide resulted in re-sensitization of SW480/tria cells to triapine, whereas no effect was observed in the parental cell line. Moreover, combination of both α5- and *αv*-specific neutralizing antibodies with triapine resulted in significant re-sensitization to triapine in SW480/tria but not the parental SW480 cell model. Together, this strongly suggests that Epac-Rap1-mediated survival signals are mediated by integrin-binding to RGD-containing ligands resulting in acquired triapine resistance. In line with this observation, several studies reported a role of altered integrin expression and especially subunit β1 in resistance against diverse cancer therapeutics in solid tumors [34]. For example, temozolomide resistance in glioblastoma [63] and vemurafenib resistance in melanoma are mediated via α5β1 integrin-dimer [64], while doxorubicin resistance in leukemia seems to be promoted by α2β1 [65]. Furthermore, taxol-resistant ovarian carcinoma patients showed a higher expression of integrin β1 [66].

When analyzing the most important downstream signaling pathways of integrins, we found a complex impact on both the MAPK and the PI3K/AKT pathways determined by phosphorylation of ERK and S6, respectively. Additionally, interesting differences between SW480 and SW480/tria cells were observed regarding Src phosphorylation. Triapine distinctly upregulated activating Src phosphorylation at Y416 specifically in the resistant subline. While, in accordance with the literature [67], Src phosphorylation was inhibited by cilengitide as single compound, combination with triapine synergistically enhanced this effect especially in SW480/tria cells to levels far above the control. Interestingly, the triapine-resistant cells exhibited a mild hypersensitivity to the Src inhibitor dasatinib. Combination of triapine with dasatinib markedly restored triapine sensitivity of SW480/tria cells. However, at lower concentrations also in SW480 cells markedly synergistic effects were observed. These data suggest that integrin-mediated Src activation is a player in both intrinsic and acquired triapine resistance. In accordance, multiple studies have suggested an important role of integrin-mediated Src hyperactivation in resistance against antineoplastic drugs but also diverse anticancer kinase inhibitors [68-71]. However, none of these studies reported upstream signaling via the Epac-Rap1 signal cascade. Additionally to Src, we also found indications that expression of the integrin-downstream GTPases RhoA and Rac might be differently affected by triapine and cilengitide in SW480 and SW480/tria cells. While triapine suppressed expression of these effector molecules, combination with cilengitide led to overexpression especially in the triapine resistant cell model. The hyperactivation of both Src phosphorylation and RhoA GTPase expression, both known to promote drug resistance [68-73], in the synergistic combination between cilengitide and triapine especially in SW480/tria cells is enigmatic. Currently, we follow the hypothesis that these effects are already reflecting escape mechanisms of the cells to survive this effective combination treatment.

Taken together, in the here presented study we established the cAMP-Epac-Rap1-integrin axis as a molecular survival mechanism to protect cancer cells from triapine-mediated cytotoxicity. Whether this mechanism is triapine-specific or might support other multi-drug resistance phenotypes needs to be established. The very broad MDR phenotype of SW480/tria cells not only against ABCB1 substrates [10] but also diverse other cytotoxic and antimetabolic drugs points into that direction. Remarkably enough, also lapatinib-resistant breast cancer as well as erlotinib-resistant non-small cell lung cancer cells harbored increased integrin β1 expression and Src phosphorylation levels [68, 69] suggesting also a contribution to tyrosine kinase inhibitor resistance. The druggability of several steps in this resistance signaling axis at the level of cAMP, Rap1, integrin and Src activation implies combination strategies as a suitable approach for overcoming triapine insensitivity of solid tumors in clinical studies.

## MATERIALS AND METHODS

### Reagents and cells

3-Aminopyridine-2-carboxaldehyde thiosemicarbazone (triapine) was synthesized at the Institute of Inorganic Chemistry of the University of Vienna [74]. H-89 dihydrochloride was purchased from Tocris Bioscience (Bristol, UK), 8-(4-Chlorophenylthio)-2'-O-methyladenosine-3',5'-cyclic monophosphate, acetoxymethyl ester (007-AM) from BioLog Life Science Institute (Bremen, Germany), cilengitide from Merck KGaA (Darmstadt, Germany) and dasatinib from Selleck Chemicals (TX, USA). Neutralizing anti-integrin *αv* (272-17E6; ab16821) and α5 (P1D6; ab78614) antibodies were purchased from Abcam (Cambridge, UK). Zoledronic acid and all other compounds were supplied by Sigma–Aldrich (MO, USA).

The human colon carcinoma-derived cell lines HCT-116 (kindly provided by Dr. Vogelstein, John Hopkins University, Baltimore, MD) and SW480 (ATCC) together with the triapine-resistant subline SW480/tria established by our group [10] were used in this study. HCT-116 was grown in McCoy's and SW480 in minimum essential medium (MEM) supplemented with 10% FCS.

### Cell viability assay

Cell viability was determined by seeding 2 x 10^4^ cells/ml on 96-well plates (100 μl/well). After a recovery period of 24 h, cells were treated with the test drugs for another 72 h. Cell viability was measured by the 3-(4,5-dimethylthiazol-2-yl)-2,5-diphenyltetrazolium bromide (MTT)-based vitality assay (EZ4U; Biomedica, Vienna, Austria) as published [75]. GraphPad Prism software was used to estimate cell viability expressed as IC_50_ values calculated from full dose–response curves (representing the drug concentrations inducing a 50% reduction of viable cells in comparison to untreated control cells cultured in parallel).

The presence of synergy was determined using the CalcuSyn software (Biosoft, Ferguson, MO, USA) according to the Chou–Talalay method [76] and expressed by the combination index (CI). CI < 0.9 represents synergism, CI = 0.9–1.1 indicates pure additivity and a CI > 1.1 points to antagonism.

### Western blot analysis

Cells were treated for 24 h at the indicated concentrations with the respective drugs. Total protein lysates were prepared, separated, and transferred onto a polyvinylidene difluoride membrane for Western blotting as described previously [75]. Following antibodies were used: Santa Cruz Biotechnology Inc (CA, USA): PDE4D (#sc-25100), 1:200; Rap1 (#sc-65) 1:1000; deprenylated Rap1 (C-17; Rap1A, #sc-65) 1:1000. Cell signaling Technology (MA, USA): phospho-PKA substrate (#9624), 1:1000; Creb (#9104), 1:1000; phospho-Creb (Ser133; #9198), 1:1000; Epac1 (#4155), 1:1000; Integrin α5 (#4705), 1:1000; Integrin *αv* (#4711), 1:1000; Integrin β1 (#9699), 1:1000; Integrin β5 (#3629), 1:1000; phospho-Erk (Thr202/Tyr204; #9101), 1:1000; Erk (#9102), 1:1000; phospho-S6 (Ser240/244; #2215), 1:1000; S6 (#2317), 1:1000; phospho-Src (Tyr416; #2101), 1:1000; Src (#2109), 1:1000; Rac (#2465), 1:1000; RhoA (67B9; #2117), 1:1000, and Vinculin (E1E9V, #13901). Sigma-Aldrich: β-actin (AC-15; #A1978), 1:1000. Secondary, horseradish peroxidase-labeled antibodies from Cell Signaling Technologies were used in working dilutions of 1:10 000.

### Cell cycle analysis

SW480 and SW480/tria cells (3 x 10^5^) were seeded in 6-well plates and allowed to recover for 24 h. Then, cells were treated with triapine and rolipram at the indicated concentrations for another 24 h at 37°C. Cells were trypsinized, treated with RNAse A (0.2 mg/ml) and stained with propidium iodide (0.01 mg/ml). Fluorescence of propidium iodide was measured by flow cytometry using FACS Calibur (Becton Dickinson, Palo Alto, CA) as described previously [77]. CellQuest Pro software (Becton Dickinson) was used to analyze the resulting DNA histograms.

### Colony formation assay

Cells were plated at a density of 3.5 x 10^3^ cells/well in 6-well plates. After 24 h, cells were treated with triapine and rolipram at the indicated concentrations and incubated for 10 days. Afterwards, cells were washed with phosphate-buffered saline (PBS), fixed with methanol for 20 min on 4°C, washed again and stained with 0.01% crystal violet. Colonies were counted and evaluated with ImageJ and GraphPad Prism software, respectively.

### Array comparative genomic hybridization (aCGH)

For aCGH analyses 4x44K oligonucleotide-based microarrays (Agilent) were used. Labeling and hybridization procedures were performed according to protocols provided by Agilent as previously described [78]. Direct aCGH was performed to detect differences between SW480 cells and normal human reference DNA and for comparison of SW480/tria to SW480 cells, indirect aCGH was performed: SW480 (instead of normal human reference DNA) was labeled with Cy3 and SW480/tria cells with Cy5.

### Whole genome gene expression analysis

Gene expression arrays were performed using 4x44K whole genome oligonucleotide-based gene expression arrays from Agilent. Labeling and hybridization procedures were performed according to the instructions provided by Agilent using the Quick Amp Labeling Kit and the Two Color Microarray-Based Gene Expression Analysis Protocol as published [78]. Feature extraction and data analysis were carried out using the Feature Extraction and Gene Spring software, respectively. For analysis in GeneSpring following parameters were used (Guided Workflow): samples were thresholded to 1, shifted to 75% percentile, and the baseline was set to median of all samples.

### Adhesion assay

Cells were treated with triapine and zoledronic acid at the indicated concentration 24 h before the adhesion assay was performed. Afterwards cells were trypsinized and washed twice with growth medium. 7.5 x 10^4^ cells/well were allowed to adhere for 1 h on 24-well plates either uncoated or coated with vitronectin (Sigma; #SRP3186; 3 μg/cm^2^), fibronectin (Millipore; #US1341576; 3 μg/cm^2^) or BSA (Roth-Lactan; #8076.2; 3 μg/cm^2^) in medium without serum. Cilengitide (1 μM) was added during the period of adhesion. Afterwards, cells were washed three-times with PBS, fixed with methanol for 20 min on 4°C, washed again and stained with 0.01% crystal violet. Colonies were counted and evaluated with ImageJ and GraphPad Prism software, respectively.

### RNA isolation and real-time PCR

Total RNA was isolated with Trizol reagent. mRNA was transcribed into cDNA and real-time polymerase chain reaction (PCR) was performed as described [79] using following primers: Integrin α5 (*ITGA5*) sense: 5’-TGCAGTGTGAGGCTGTGTACA-3’ and antisense: 5’-GTGGCCACCTGACGCTCT-3’; Integrin *αv* (*ITGAV*) sense: 5’-AATCTTCCAATTGAGGATATCAC-3’ and antisense: 5’-AAAACAGCCAGTAGCAACAAT-3’. *β-actin* sense: 5’-GGATGCAGAAGGA GATCACTG-3’ and antisense: 5’-CGATCCACACGGAGTACTTG-3’. β-actin served as a housekeeping control.

### Rap1 activation assay

2 x 10^7^ cells were seeded in 150 cm^2^ flasks. After 24 h recovery time, cells were harvested, lysed and treated according to manufacturer's protocol (Cell Signaling Technology; Active Rap1 Detection Kit, #8818).

### Xenograft experiments and immunohistochemistry

Six to eight week old male CB-17 scid/scid (SCID) mice were purchased from Harlan Laboratories and kept in groups of four per cage in a pathogen-free environment. 1 x 10^6^ SW480 or SW480/tria cells diluted in serum-free RPMI were injected subcutaneously into the right flank of the animals. Tumor size (caliper measurement) was assessed every second day. Tumor volume was calculated using the formula (length x width^2^)/2. All experiments were authorized by the Ethics committee at the Medical University Vienna and carried out according to the Austrian and the Federation of Laboratory Animal Science Associations (FELASA). For histological evaluations, 3 μm formalin-fixed and paraffin-embedded tumor sections were deparaffinised and rehydrated. Sections were stained with hematoxylin and eosin (H&E), Ki-67 (clone MiB-1; DAKO, Glostrup, Denmark) 1:100, and integrin β1 (CD29, #610467; BD Bioscience, NJ, USA) 1:400 as described previously [80].

## SUPPLEMENTARY MATERIALS FIGURES


