# Supplementary material for: Loss of phosphodiesterase 4D mediates acquired triapine resistance via Epac-Rap1-Integrin signaling
Source: Oncotarget. 2016 Sep 2;7(51):84556–74. doi: 10.18632/oncotarget.11821 (PMC5356681; doi:10.18632/oncotarget.11821)
Supplement: Supplementary file 1 [file oncotarget-07-84556-s001.pdf]

Loss of phosphodiesterase 4D mediates acquired triapine resistance via Epac-Rap1-Integrin signaling

SUPPLEMENTARY FIGURES

A

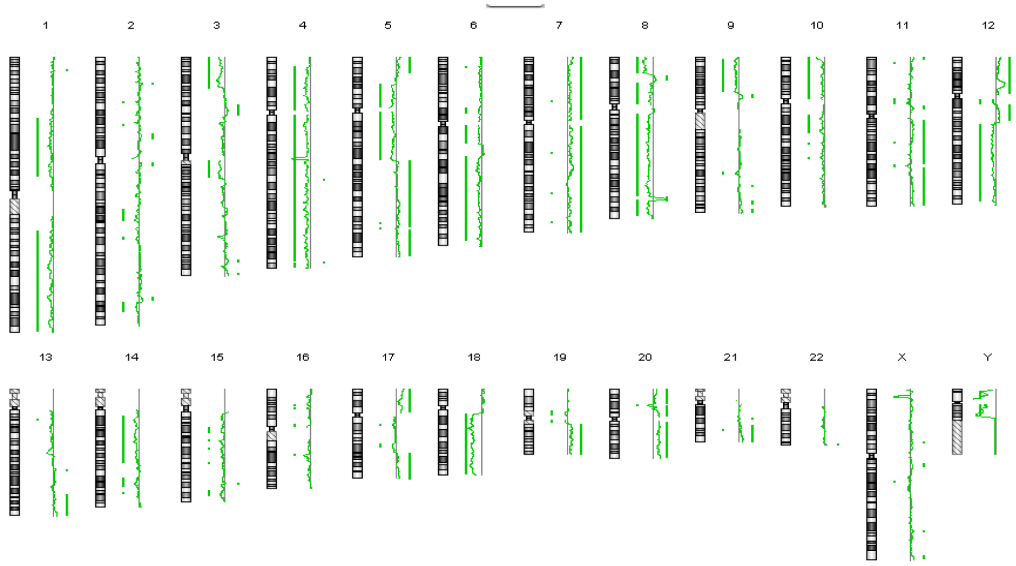

B

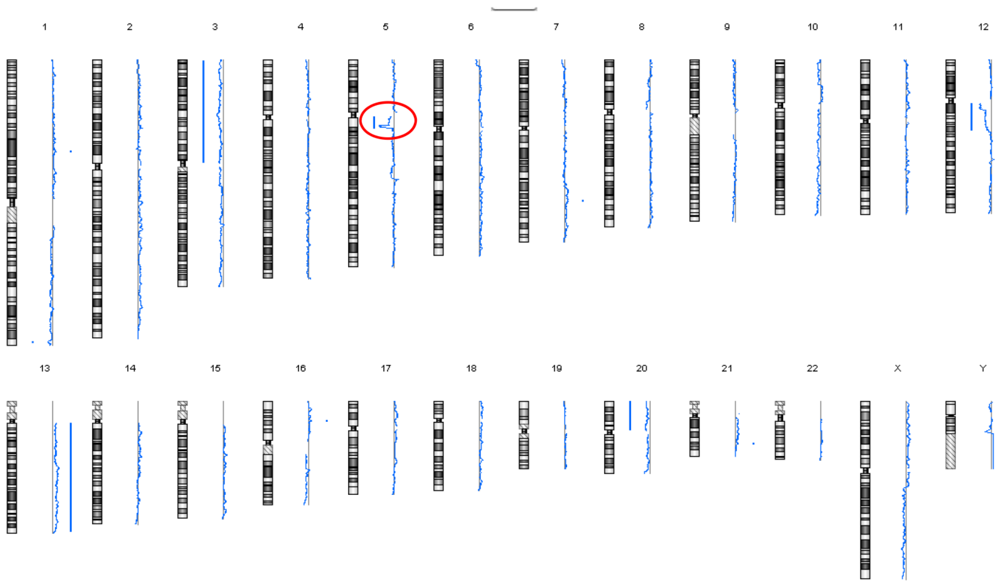

Supplementary Figure S1: Genomic changes of SW480 colon cancer cells before and after selection for acquired triapine resistance. A. Gene dose alterations in SW480 colon cancer cells were investigated by aCGH. Data are depicted using Agilent Genomic Workbench software (Version 7). **B.** Using an indirect aCGH approach, DNA from SW480/tria was compared to that of SW480 cells. The red circle indicates the locus of the *PDE4D* gene on chromosome 5q12.

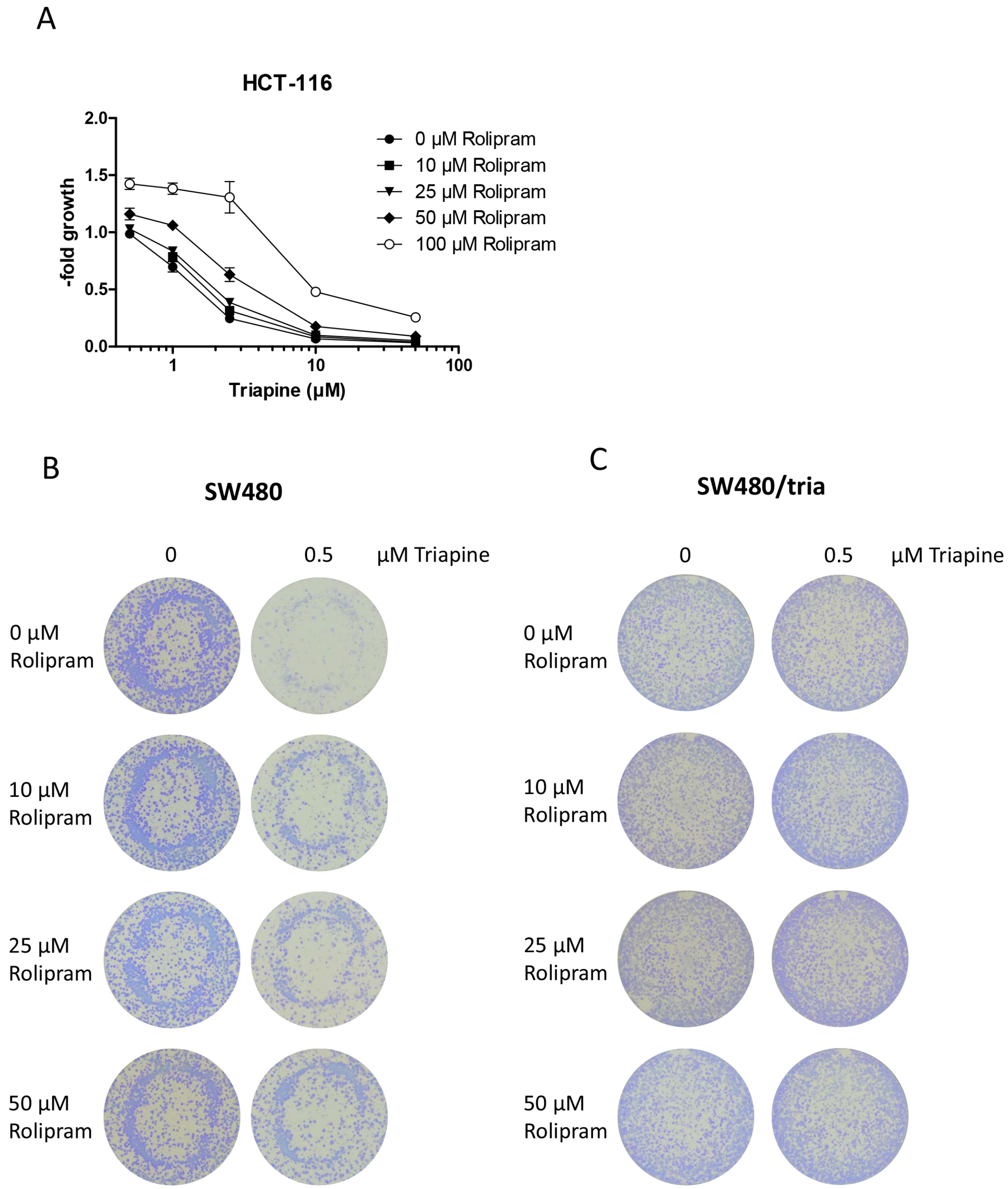

Supplementary Figure S2: Impact of PDE4D inhibition on triapine response in human colon cancer cells. A. Cell viability of HCT-116 cells treated for 72 h with the PDE4D inhibitor rolipram at the indicated concentrations alone and in combination with 1 µM triapine was determined by MTT assay. The values given are means and SD of one representative experiment out of three performed in triplicate. **B** and **C.** For colony formation assays, SW480 (B) and SW480/tria cells (C) were treated with the indicated concentrations of triapine in combination with rolipram (PDE4D inhibitor). After 10 days drug exposure, cells were stained with crystal violet and clone formation quantified microscopically.

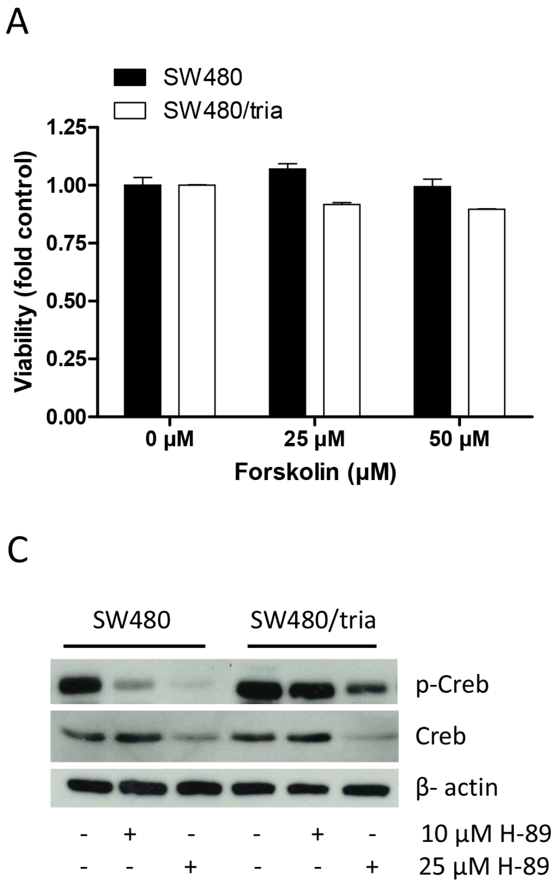

Supplementary Figure S3: The PKA-Creb signaling axis is not involved in triapine resistance. **A** and **B.** SW480 and SW480/tria cells were treated with the indicated concentrations of forskolin (adenylate cyclase activator; A) or H-89 (PKA inhibitor; B). After 72 h treatment, cell viability was determined by MTT assay. The values given are means and SD of three independent experiments performed in triplicate. **C.** To confirm PKA inhibition by H-89, SW480 and SW480/tria cells were treated with the indicated concentrations and total protein extracts were isolated. Activating phosphorylation of Creb was determined by Western blotting. β-actin was used as loading control.

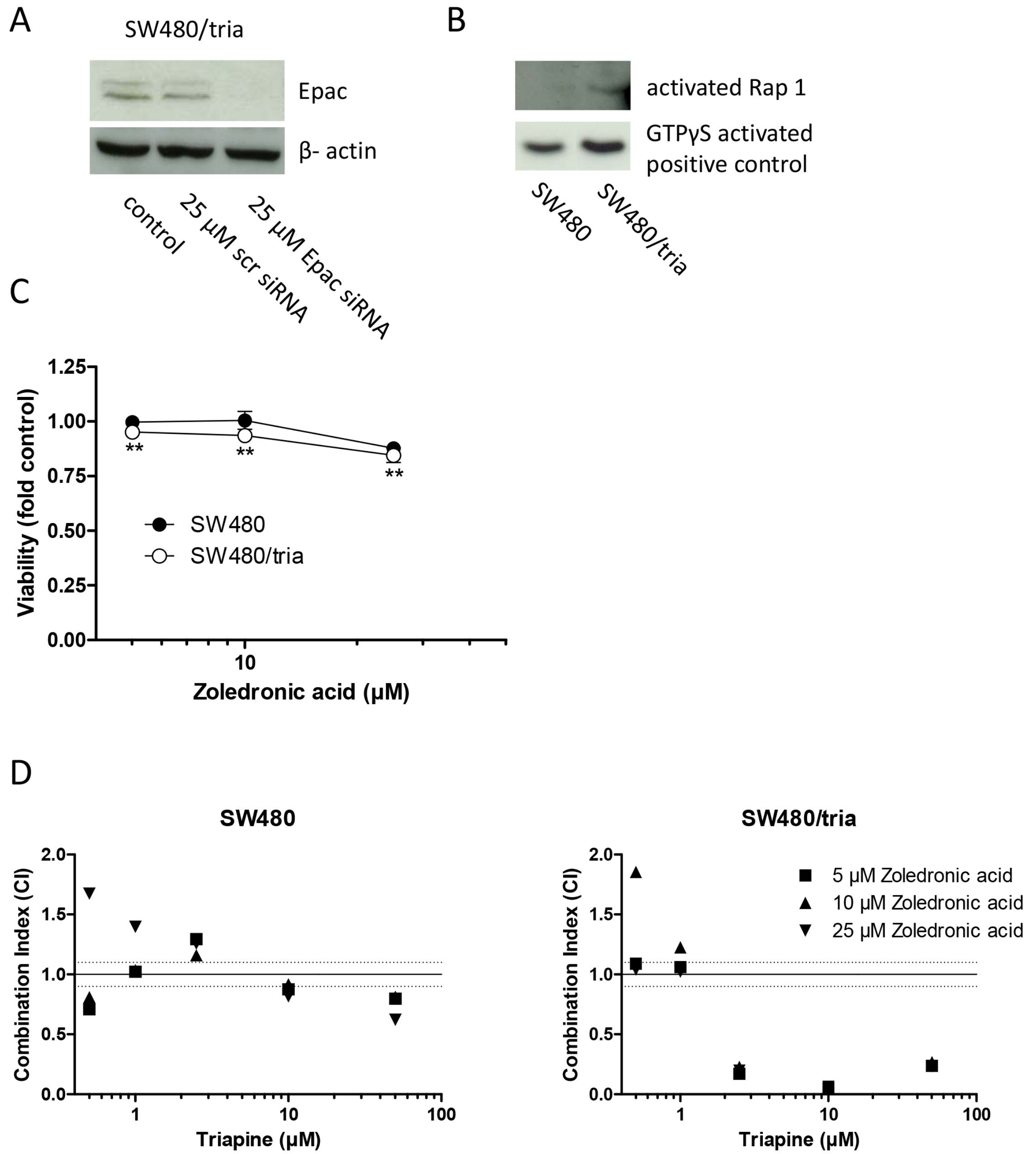

Supplementary Figure S4: The Epac-Rap1-integrin axis contributes to acquired triapine resistance. A. To confirm *Epac* knock-down efficacy by siRNA, SW480/tria cells were treated with the indicated concentrations of *Epac* siRNA and scrambled (scr) siRNA for 48 h and total protein extracts were isolated. Epac protein expression was determined by Western blotting and β-actin was used as loading control. **B.** Activated Rap1 was detected by the pull-down assay in SW480 and SW480/tria cells. As positive control SW480 and SW480/tria cells were treated with GTPγS. Total protein extracts of SW480 vs. SW480/tria cells was analysed by Western blotting. **C.** SW480 and SW480/tria cells were treated with the indicated concentrations of zoledronic acid (inhibiting Rap1 prenylation). After 72 h treatment cell viability was determined by MTT assay. The values given are means and SD of three independent experiments performed in triplicates. Statistical analysis was performed by two-way ANOVA (**$P < 0.01$). **D.** Combination indices (CI) for the 72 h analyses shown in Figure 4E were calculated using CalcuSyn software. CI < 0.9, CI = 0.9 - 1.1 or CI > 1.1 represent synergism, additive effects and antagonism, respectively.

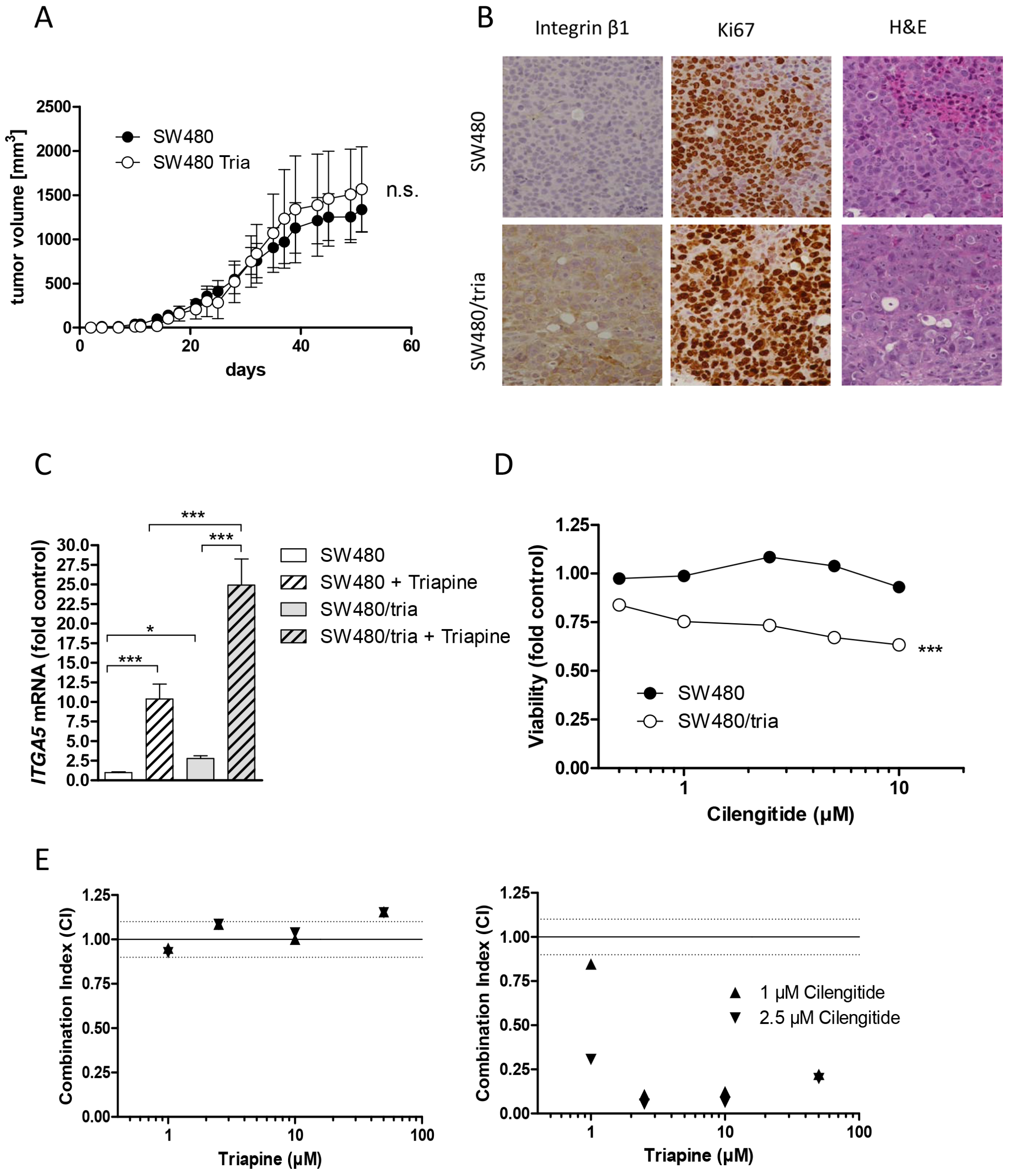

Supplementary Figure S5: Tumorigenicity of SW480 and SW480/tria cells. A. Cells as indicated (1x10[6]/mouse) were xenografted subcutaneously into four SCID mice per group. Tumor size was measured with caliper every second day. **B.** Immunhistochemical detection of integrin β1, Ki67 and H&E staining of SW480 and SW480/tria xenografts. **C.** Integrin α5 mRNA (*ITGA5*) expression was determined by real-time PCR in SW480 and SW480/tria cells treated with 50 μM triapine (+Triapine) for 24 h. Expression levels were normalized to *β-actin* employed as housekeeping gene and given normalized to untreated SW480 cells (control). Statistical analysis was performed by one-way ANOVA (*$P< 0.05$; ***$P < 0.001$). **D.** SW480 and SW480/tria cells were treated with the indicated concentrations of cilengitide (integrin inhibitor). After 72 h treatment, cell viability was determined by MTT assay. The values given are means and SD of three independent experiments performed in triplicates. Statistical analysis was performed by two-way ANOVA (***$P < 0.01$). **E.** Combination indices (CI) for the 72 h analyses shown in Figure 5C were calculated using CalcuSyn software. CI < 0.9, CI = 0.9 - 1.1 or CI > 1.1 represent synergism, additive effects and antagonism, respectively.

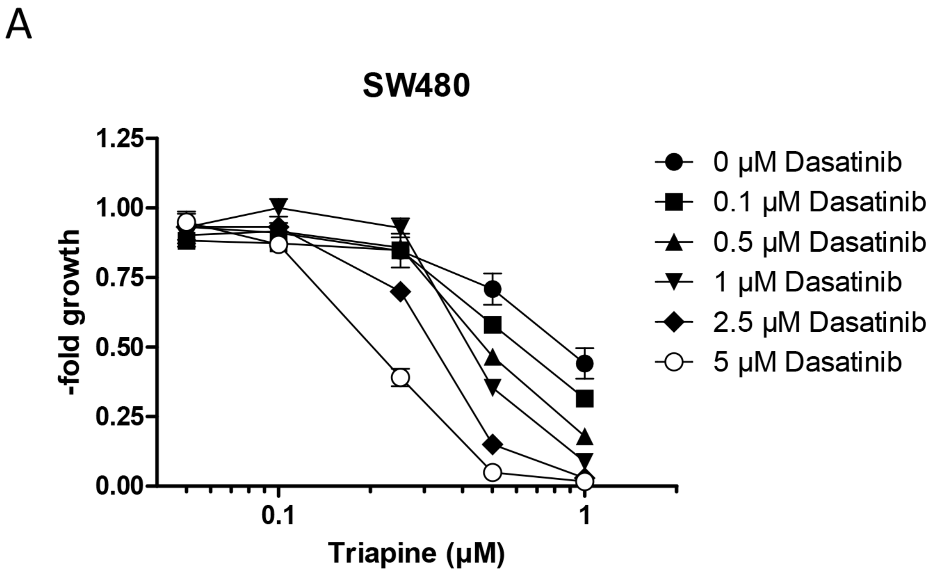

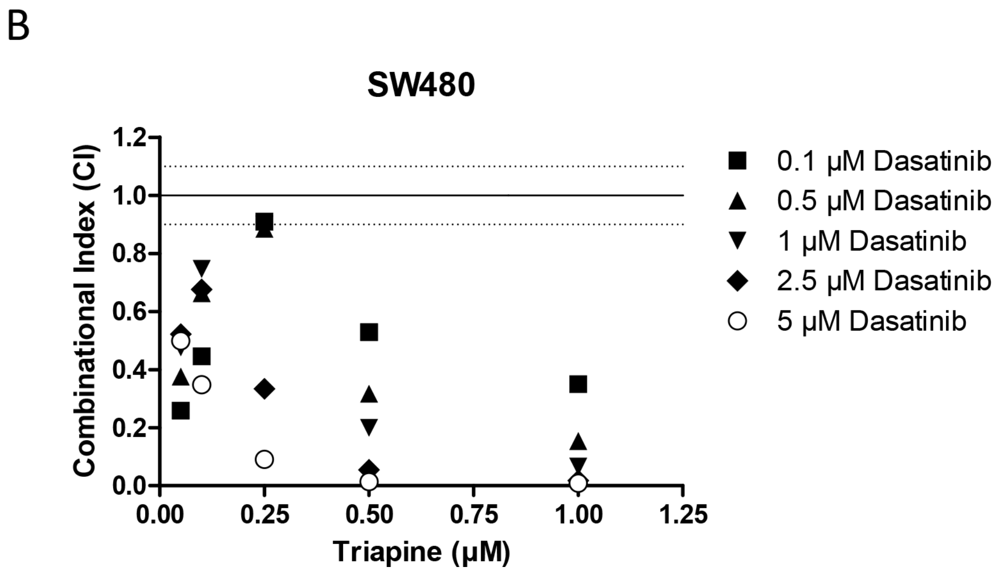

Supplementary Figure S6: Src inhibition results in re-sensitization to triapine in SW480 cells. A. SW480 cells were treated with the indicated low concentrations of triapine alone and in combination with dasatinib (Src inhibitor). After 72 h treatment, cell viability was determined by MTT assay. The values given are means and SD of one representative experiment out of three performed in triplicate. **B.** Combination indices (CI) for the 72 h analyses shown in Supplementary Figure 6A were calculated using CalcuSyn software. CI < 0.9, CI = 0.9 - 1.1 or CI > 1.1 represent synergism, additive effects and antagonism, respectively.
